# Clinical Studies on the Supplementation of Manufactured Human Milk Oligosaccharides: A Systematic Review

**DOI:** 10.3390/nu15163622

**Published:** 2023-08-17

**Authors:** Yannik Bernd Schönknecht, María Virginia Moreno Tovar, Stina Rikke Jensen, Katja Parschat

**Affiliations:** 1 Chr. Hansen HMO GmbH, 53619 Rheinbreitbach, Germany; dekapa@chr-hansen.com; 2 Chr. Hansen A/S, 2970 Hoersholm, Denmark; vrgmorenotovar@hotmail.com (M.V.M.T.); dkstaj@chr-hansen.com (S.R.J.)

**Keywords:** infant formula, human milk oligosaccharide, clinical trial, growth, tolerance, safety, microbiome, immunity

## Abstract

Human milk oligosaccharides (HMOs) are a major component of human milk. They are associated with multiple health benefits and are manufactured on a large scale for their addition to different food products. In this systematic review, we evaluate the health outcomes of published clinical trials involving the supplementation of manufactured HMOs. We screened the PubMed database and Cochrane Library, identifying 26 relevant clinical trials and five publications describing follow-up studies. The clinical trials varied in study populations, including healthy term infants, infants with medical indications, children, and adults. They tested eight different HMO structures individually or as blends in varying doses. All trials included safety and tolerance assessments, and some also assessed growth, stool characteristics, infections, gut microbiome composition, microbial metabolites, and biomarkers. The studies consistently found that HMO supplementation was safe and well tolerated. Infant studies reported a shift in outcomes towards those observed in breastfed infants, including stool characteristics, gut microbiome composition, and intestinal immune markers. Beneficial gut health and immune system effects have also been observed in other populations following HMO supplementation. Further clinical trials are needed to substantiate the effects of HMO supplementation on human health and to understand their structure and dose dependency.

## 1. Introduction

Breastfeeding is widely recognized as the optimal form of nutrition during early life, providing numerous immediate and long-term health benefits for infants [1,2]. Human milk is a complex biological system containing many bioactive components whose function goes beyond nutrition [3,4,5]. A special characteristic of human milk is the high content of complex oligosaccharides, also known as human milk oligosaccharides (HMOs). These are the third most abundant solid component of breastmilk after lactose and lipids, and more than 150 structures have been identified thus far [6]. All HMOs contain lactose at the reducing end and can be classified according to their structure as fucosylated, neutral, non-fucosylated, or sialylated (acidic) [7,8]. A small number of HMO structures may deviate from this general blueprint [9].

**Figure 1:**
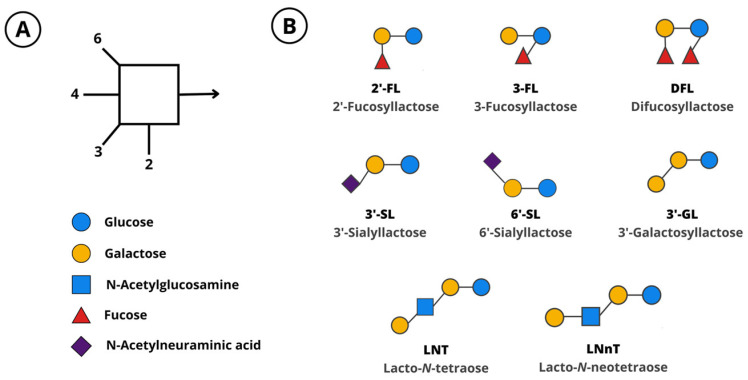
(**A**) Monosaccharides and linkages that compose human milk oligosaccharides. (**B**) Manufactured oligosaccharides. 2′-FL, 2′-fucosyllactose; 3-FL, 3-fucosyllactose; 3′-GL, 3′-galactosyllactose; 3′-SL, 3′-sialyllactose; 6′-SL, 6′-sialyllactose; DFL, difucosyllactose; LNT, lacto-*N*-tetraose; LNnT, lacto-*N*-*neo*tetraose.

HMOs are associated with beneficial health outcomes in infants and have become the subject of intense research. They are mostly resistant to digestion in the gut and are absorbed in only low amounts [10,11]. They naturally act as prebiotics, which can be utilized selectively by intestinal bacteria to support the establishment of a balanced gut microbiome [12,13,14]. The microbial fermentation of HMOs results in the formation of bacterial metabolites, such as short-chain fatty acids (SCFAs) and aromatic lactic acids, which have local and systemic effects [15,16]. HMOs also act as decoy glycan structures that pathogens bind to, preventing their interaction with cell-surface receptors [17,18]. Additionally, observational and preclinical studies suggest that HMOs have direct, microbiome-independent effects. These include interactions with immune cells, thereby modulating the immune system [17,19,20], maturation of the intestinal glycocalyx [21], and the promotion of neurodevelopment [7,22,23]. HMOs have also been detected in the serum of pregnant women, in cord blood, and in amniotic fluid, suggesting a role in fetal development [24]. Advances in biotechnology now allow the large-scale production of HMOs that are chemically and structurally identical to those found in human milk. Several HMOs can be manufactured by bacterial fermentation [25,26] or enzymatic conversion [27]. Manufactured HMOs include 2′-fucosyllactose (2′-FL), 3-fucosyllactose (3-FL), difucosyllactose (DFL), lacto-*N*-tetraose (LNT), lacto-*N*-*neo*tetraose (LNnT), 3′-sialyllactose (3′-SL), and 6′-sialyllactose (6′-SL). Some oligosaccharides that are identical to those in human milk, such as 3′-galactosyllactose (3′-GL), can be produced by the fermentation of galactooligosaccharides (GOS) [28]. The structure of these oligosaccharides can be found in Figure 1.

Today, HMOs for supplementing infant formula are produced by using engineered suitable host strains such as *Escherichia coli*, *Corynebacterium glutamicum,* or *Saccharomyces cerevisiae* as cell factories. These bacteria synthesize HMOs from simple carbon sources and lactose and secrete the product into the fermentation medium. The product is retrieved from the cell-free fermentation supernatant and purified to a powder that complies with the regulations for specific food segments. These manufactured HMOs can therefore be used to supplement foods, such as infant formulas, driving preclinical and clinical research on the effects of HMOs in the diet [29,30].

To the best of our knowledge, this is the first systematic review covering the use of manufactured HMOs as food supplements in humans. Earlier reviews have focused on the addition of HMOs to infant formula products [30,31,32,33] or have included studies involving bovine milk oligosaccharides [29]. Another existing review focused entirely on HMO supplements for the management of irritable bowel syndrome (IBS) in adults [34]. Here, we provide a comprehensive overview of clinical studies conducted in populations of all ages that have supplemented manufactured HMOs to different food products and summarize the evidence on health outcomes and the current state of clinical research.

## 2. Materials and Methods

We conducted a systematic literature search between October and November 2022 to identify relevant studies. We screened PubMed (https://pubmed.ncbi.nlm.nih.gov) by applying the search term “human milk oligosaccharide AND clinical trial”. Titles and abstracts were evaluated based on predefined inclusion and exclusion criteria (Table 1). A second literature search of the Cochrane Library (https://www.cochranelibrary.com) was conducted by applying the same search term and criteria. Eligible abstracts were evaluated by screening the full text. Additional studies were found by computer-assisted manual searches. Following the selection, relevant data were extracted from each publication independently by two authors and tabulated.

## 3. Results

### 3.1. Overview of the Literature Search

**Figure 2:**
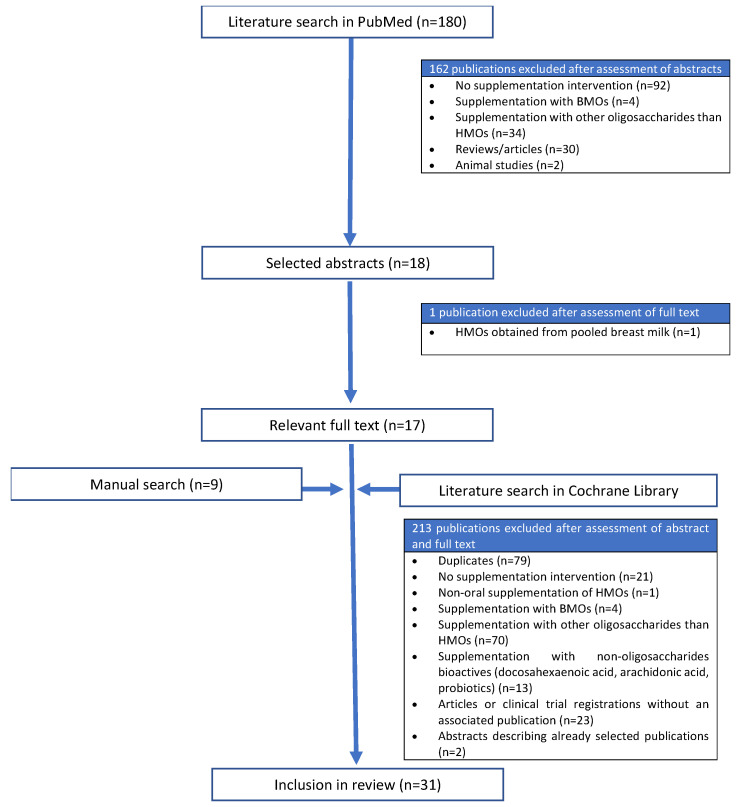
Flow diagram showing the literature screening process. BMOs, bovine milk oligosaccharides; HMOs, human milk oligosaccharides.

The initial literature search of PubMed identified 180 publications (Figure 2). After screening the titles and abstracts, 162 publications were excluded. We analyzed the full text of the remaining 18 publications and excluded one further study because the study product was supplemented with a pool of HMOs extracted from human breast milk [35]. The initial literature search of the Cochrane Library identified 218 publications. After screening the titles and abstracts, 213 publications were excluded. Among these, two of the excluded articles were conference contributions that summarized the results of already selected publications [36,37], and one was a clinical study describing the intranasal application of HMOs [38]. In addition to the 17 selected studies from PubMed and the five from the Cochrane Library, nine more articles that met our criteria were identified by manual searching, bringing the total number of included studies to 31.

### 3.2. Description of Studies

Among the 31 accepted publications, 26 described clinical trials and 5 reported follow-up studies. Twelve of the clinical studies involved healthy term infants, as well as four of the follow-up publications (Table 2). Five studies involved infants with medical conditions, of which four included infants with diagnosed or suspected allergies, and one included preterm infants (Table 3). One study involved children 1–2.5 years of age, and another included children 6–12 years of age that were overweight or obese (Table 4). Finally, eight studies involved adults (>18 years of age), of which two focused on healthy individuals, three on individuals with IBS (plus one follow-up study), and two focused on individuals diagnosed with *Helicobacter pylori* infection (Table 5).

### 3.3. Investigated HMOs, Doses, and Period of Supplementation

Eight different manufactured HMO structures have been studied in 26 clinical studies: 2′-FL, 3-FL, DFL, LNT, LNnT, 3′-GL, 3′-SL, and 6′-SL. The most frequently studied HMO is 2′-FL, which has been investigated in 22 clinical trials (Table 6).

**Table 1:** Inclusion and exclusion criteria for the screening of studies in this systematic review.

Inclusion Criteria	Exclusion Criteria
Clinical trials Oral supplementation of manufactured HMOs as intervention (individually, in blends, or in combination with other bioactives) HMOs supplemented as pure product or another food product containing manufactured HMOs Individuals of any age Healthy individuals or those with medical conditions Publication available in English language	Non-clinical studies Intake of HMOs from breastfeeding Supplementation of HMO pools isolated from human or bovine milk Supplementation of prebiotics other than HMOs Probiotics or other bioactives as sole intervention Interventions on mothers to evaluate offspring Abstracts duplicating data from other publications

**Table 2:** Clinical trials that investigated human milk oligosaccharides supplementation in healthy term infants.

Reference	Study Population	Study Design	Intervention Groups	Comparison	Duration of Intervention	Key Findings
[39]	Healthy term infants aged 6–24 months (n = 228)	Randomized, double-blind multicenter, controlled trial	0.2 g/L LNnT (n = 115)	Control formula without oligosaccharides (n = 113)	16 weeks	- Safe and well-tolerated - No effect on oropharyngeal colonization with *Streptococcus pneumoniae*
[40]	Healthy term infants < 5 days at inclusion (n = 420)	Randomized, double-blind, multicenter, controlled trial	- Intervention 1: 0.2 g/L 2′-FL + 2.2 g/L GOS (n = 104) - Intervention 2: 1.0 g/L 2′-FL + 1.4 g/L GOS (n = 109)	- Control formula: 2.4 g/L GOS (n = 101) - BF reference group (n = 106)	17 weeks	- Safe and well tolerated - Supports normal, age-appropriate growth - Similar relative absorption and relative excretion of 2′-FL in intervention and BF groups at DOL 42 and 119
[41]	Follow-up analysis of [40]	-	- Subpopulation from intervention 1: 0.2 g/L 2′-FL + 2.2 g/L GOS (n = 76) - Subpopulation from intervention 2: 1.0 g/L 2′-FL + 1.4 g/L GOS (n = 78)	- Subpopulation from control formula: 2.4 g/L GOS (n = 75) - Subpopulation from BF reference group (n = 86)	-	- Similar cytokine profile and ex vivo stimulation of PBMCs in intervention and BF groups at 6 weeks
[42]	Healthy term infants < 8 days at inclusion (n = 119)	Randomized, double-blind, multicenter, controlled trial	0.2 g/L 2′-FL + 2 g/L scFOS (n = 46)	- Control formula without oligosaccharides (n = 42) - BF reference group (n = 43)	35 days	- Well tolerated - Supports normal, age-appropriate growth
[43]	Healthy term infants < 14 days at inclusion (n = 175)	Randomized, double-blind, multicenter, controlled trial	1.0 g/L 2′-FL + 0.5 g/L LNnT (n = 88)	- Control formula without oligosaccharides (n = 87) - BF reference group (n = 38)	6 months (HMO intervention) + follow-up up to 12 months of age (no HMO intervention)	- Safe and well tolerated - Supports normal, age-appropriate growth - Reduced parent-reported cases of bronchitis and medication use during the first 12 months of life - Reduced incidence of infantile colic at 4 months
[44]	Healthy term infants 14 ± 5 days at inclusion (n = 79)	Randomized, double-blind, multicenter, controlled trial	0.25 g/L 2′-FL + *Bifidobacterium animalis* ssp. *lactis*. Partially hydrolyzed formula (n = 39)	Control formula without oligosaccharides + *Bifidobacterium animalis* ssp. *lactis* (n = 40)	6 weeks	- Safe and well tolerated - Supports normal, age-appropriate growth - Trend of reduced number of reported infections (not statistically significant)
[45]	Follow-up analysis of [43]	-	Subpopulation from intervention: 1.0 g/L 2′-FL + 0.5 g/L LNnT (n = 58)	- Subpopulation from control formula without oligosaccharides (n = 64) - Subpopulation from BF reference group (n = 35)	-	- Gut microbiome composition shifted towards BF infants - Reduced use of antibiotics during the first 12 months of life
[46]	Healthy term infants 7 days to 2 months at inclusion (n = 207)	Non-randomized, open-label, multicenter trial	1.0 g/L 2′-FL + 0.5 g/L LNnT. Partially hydrolyzed formula (n = 66)	- Mixed group: HMO formula + BF - BF reference group (n = 45)	8 weeks	- Safe and well tolerated - Supports normal, age-appropriate growth
[47]	Healthy term infants < 14 days at inclusion (n = 276)	Randomized, double-blind, multicenter, controlled trial	1.0 g/L 2′-FL + 7.2 g/L GOS + 0.8 g/L FOS, including 0.015 g/L 3′-GL. Partially hydrolyzed formula (n = 108)	- Control formula without 2′-FL and 3′-GL, with 7.2 g/L GOS + 0.8 g/L FOS (n = 107) - BF reference group (n = 61)	17 weeks	- Safe and well tolerated - Supports normal, age-appropriate growth
[48]	Follow-up analysis [43]	-	-	-	-	- Gut microbiome composition shifted towards BF infants - Effect on fecal biomarkers at 3 months
[49]	Healthy term infants < 14 days at inclusion (n = 341)	Randomized, double-blind, multicenter, controlled trial	5.75 g/L HMOs: 2.99 g/L 2′-FL + 0.75 g/L 3-FL + 1.5 g/L LNT + 0.23 g/L 3′-SL + 0.28 g/L 6′-SL (n = 113)	- Control formula without oligosaccharides (n = 112) - BF reference group (n = 116)	16 weeks (HMO intervention) + 8-week voluntary follow-up (HMO intervention)	- Safe and well tolerated - Supports normal, age-appropriate growth - Stool characteristics shifted towards BF infants
[50]	Healthy term infants < 14 days at inclusion (n = 289)	Randomized, double-blind, multicenter, controlled trial	1.0 g/L 2′-FL + *Limosilactobacillus reuteri* (n = 144)	- Control formula without oligosaccharides + *Limosilactobacillus reuteri* (n = 145) - BF reference group (n = 60)	6 months	- Safe and well tolerated - Supports normal, age-appropriate growth - Gut microbiome composition shifted towards BF infants
[51]	Healthy term infants < 21 days at inclusion (n = 535)	Randomized, double-blind, multicenter, controlled trial	- Intervention 1: 1.5 g/L HMOs: 0.87 g/L 2′-FL + 0.10 g/L DFL + 0.29 g/L LNT + 0.11 g/L 3′-SL + 0.14 g/L 6′-SL (n = 153) - Intervention 2: 2.5 g/L HMOs: 1.45 g/L 2′-FL + 0.14 g/L DFL + 0.48 g/L LNT + 0.18 g/L 3′-SL + 0.24 g/L 6′-SL (n = 158)	- Control formula without oligosaccharides (n = 155) - BF reference group (n = 69)	6 months	- Gut microbiome composition shifted towards BF infants - Effect on fecal biomarkers at 3 and 6 months
[52]	Healthy term infants < 14 days at inclusion (n = 363)	Randomized, double-blind, multicenter, controlled trial	5.75 g/L HMOs: 3.0 g/L 2′-FL + 0.80 g/L 3-FL + 1.5 g/L LNT + 0.20 g/L 3′-SL + 0.30 g/L 6′-SL (n = 130)	- Control formula without oligosaccharides (n = 129) - BF reference group (n = 104)	4 months	- Safe and well tolerated - Supports normal, age-appropriate growth - Stool characteristics shifted towards BF infants - Reduced number of visits to healthcare professionals
[53]	Follow-up analysis [43]	-	-	-	-	- Effect on gut-microbiome fecal co-metabolite profile
[54]	Healthy term infants < 28 days at inclusion (n = 221)	Randomized, double-blind, multicenter, controlled trial	1.0 g/L 2′-FL + FOS (not specified) (n = 66)	- Control formula + GOS + FOS (not specified) (n = 66) - BF reference group (n = 89)	16 weeks	- Safe - Supports normal, age-appropriate growth - Gut microbiome composition shifted towards BF infants - Increased microbial metabolic capacity to utilize fucosylated HMOs

2′-FL, 2′-fucosyllactose; 3-FL, 3-fucosyllactose; 3′-GL, 3′-galactosyllactose, 3′-SL, 3′-sialyllactose; 6′-SL, 6′-sialyllactose; BF, breastfed; DFL, difucosyllactose; DOL, day of life; FOS, fructooligosaccharides; GOS, galactooligosaccharides; HMO, human milk oligosaccharide; LNT, lacto-*N*-tetraose; LNnT, lacto-*N*-*neo*tetraose; PBMCs, peripheral blood mononuclear cells; scFOS, short-chain fructooligosaccharides.

**Table 3:** Clinical trials that investigated human milk oligosaccharide supplementation in infants with medical indications.

Reference	Study Population	Study Design	Intervention Groups	Comparison	Duration of Intervention	Key Findings
[55]	Infants and children between 2 months and 4 years with diagnosed CMPA (n = 67)	Randomized, double-blind, placebo-controlled food challenge procedure (DBPCFC) and open-label challenge	1.0 g/L 2′-FL + 0.5 g/L LNnT. Extensively hydrolyzed formula (n = 36 in DBPCFC, n = 62 in open-label challenge)	Control formula without oligosaccharides (n = 31, only in DBPCFC)	1 week	- Safe and well tolerated - Confirmed hypo-allergenicity
[56]	Infants < 60 days at inclusion with suspected food protein allergy/sensitivity (n = 48)	Non-randomized, multicenter, single-arm trial	0.2 g/L 2′-FL. Extensively hydrolyzed formula (n = 48)	-	60 days	- Safe and well tolerated - Supports normal, age-appropriate growth - Improved and resolved allergy symptoms
[57]	Term infants aged 0–6 months diagnosed with CMPA (n = 194)	Randomized, double-blind, multicenter, controlled trial	1.0 g/L 2′-FL + 0.5 g/L LNnT. Extensively hydrolyzed formula (n = 94)	Control formula without oligosaccharides (n = 96)	4 months (HMO intervention) + follow-up up to 12 months of age (no HMO intervention)	- Safe and well tolerated - Supports normal, age-appropriate growth - No effect on allergy symptoms - Reduced frequency of URTIs and risk of otitis media - Reduced use of antipyretics between 4 months and 12 months
[58]	Term infants aged 1–8 months diagnosed with moderate-to-severe CMPA (n = 32)	Non-randomized, open-label, multicenter, single-arm trial	1.0 g/L 2′-FL + 0.5 g/L LNnT. Amino acid-based formula (n = 32)	-	4 months + voluntarily up to 12 months of age (HMO intervention)	- Safe and well tolerated - Supports normal, age-appropriate growth - Stool characteristics and gut microbiome composition shifted towards BF infants - Effect on SCFA concentrations in feces - Improved allergy symptoms except for two infants, who experienced adverse events associated with the study formula
[59]	Preterm infants with very low birth weight < 1700 g (n = 86)	Randomized, double-blind, multicenter, controlled trial	0.34 g/kg/d 2′-FL + 0.034 g/kg/d LNnT (n = 43)	Glucose placebo (0.140 g/kg/d) (n = 43)	-	- Safe and well tolerated - Increased length-for-age z-score at day 14 and 21 before full enteral feeding - Increased mean head circumference gain at day 21 before full enteral feeding

2′-FL, 2′-fucosyllactose, CMPA, cows’ milk protein allergy; HMO, human milk oligosaccharide; LNnT, lacto-*N*-*neo*tetraose; URTI, upper respiratory tract infection.

**Table 4:** Clinical trials that investigated human milk oligosaccharide supplementation in children populations.

Reference	Study Population	Study Design	Intervention Groups	Comparison	Duration of Intervention	Key Findings
[60]	Healthy children aged 1–2.5 years (n = 461)	Randomized, double-blind, single-center, controlled trial	-Intervention 1: Formula with 3.0 g/L 2′-FL (n = 114) - Intervention 2: Formula with 3.0 g/L 2′-FL + lactoferrin, immunoglobulins, TGF-β and milk fat (n = 114)	- Formula with no supplements (n = 114) - Formula with lactoferrin, immunoglobulins, TGF-β and milk fat (n = 114)	6 months	- Safe and well tolerated - Supports normal, age-appropriate growth - Reduced number of days with hard stools - Effect on reported duration of URTIs - No effect on gut and nasal microbiome composition
[61]	Overweight/obese children aged 6–12 years (n = 75)	Randomized, double-blind, single-center, controlled trial	- Intervention 1: 4.5 g/d 2′-FL (n = 25) - Intervention 2: 4.5 g/d 2′-FL + LNnT at 4:1 ratio (n = 25)	4.5 g/d glucose placebo (n = 25)	8 weeks	- Safe and well tolerated - Effect on gut microbiome composition - No effect on stool characteristics, blood, and fecal markers

Abbreviations: 2′-FL, 2′-fucosyllactose; BF, breastfed; LNnT, lacto-*N*-*neo*tetraose; TGF-β, transforming growth factor β; URTI, upper respiratory tract infection.

**Table 5:** Clinical trials that investigated human milk oligosaccharides supplementation in adult populations.

Reference	Study Population	Study Design	Intervention Groups	Comparison	Duration of Intervention	Key Findings
[62]	Adults with diagnosed *H. pylori* infection (n = 6)	Open-label study	10 g/d 3′-SL (n = 6)	-	1 day	- No effects on *H. pylori* infection - Well tolerated - No effects on blood markers
[63]	Adults with diagnosed *H. pylori* infection (n = 65)	Randomized, double-blind, placebo-controlled trial	Intervention 1: 10 g/d 3′-SL (n = 17) Intervention 2: 20 g/d 3′-SL (n = 22)	Placebo (not specified, n = 21)	4 weeks	- No effects on *H. pylori* infection - Well tolerated
[64]	Healthy adults (n = 100)	Randomized, double-blind, single-center, controlled trial	- Intervention 1: 5 g/d 2′-FL (n = 10) - Intervention 2: 10 g/d 2′-FL (n = 10) - Intervention 3: 20 g/d 2′-FL (n = 10) - Intervention 4: 5 g/d LNnT (n = 10) - Intervention 5: 10 g/d LNnT (n = 10) - Intervention 6: 20 g/d LNnT (n = 10) - Intervention 7: 5 g/d 2′-FL + LNnT at 2:1 ratio (n = 10) - Intervention 8: 10 g/d 2′-FL + LNnT at 2:1 ratio (n = 10) - Intervention 9: 20 g/d 2′-FL + LNnT at 2:1 ratio (n = 10)	2 g/d glucose placebo (n = 10)	2 weeks	- Safe and well tolerated - Reported gastrointestinal symptoms in some higher-dose intervention groups - Effect on gut microbiome composition - No effect on blood and fecal markers
[65]	Adults with IBS (n = 61)	Randomized, double-blind, single-center, controlled trial	- Intervention 1: 5 g/d 2′-FL + LNnT at 4:1 ratio (n = 20) - Intervention 2: 10 g/d 2′-FL + LNnT at 4.1 ratio (n = 20)	5 g/d glucose placebo (n = 21)	4 weeks supplementation + 4-week follow-up (no HMO intervention)	- Well tolerated - No worsening of IBS symptoms - No effect on gut microbiome composition
[66]	Follow-up analysis of [65]	-	-	-	-	- Effect on microbiome composition in fecal and mucosal colonic biopsies samples - Effect on fecal and plasma biomarkers
[67]	Adults with IBS (n = 317)	Open-label, multicenter, single-arm trial	4 g/d 2′-FL + 1 g/d LNnT (n = 317)	-	12 weeks	- Safe and well tolerated - Improved IBS symptoms - Effect on stool characteristics
[68]	Adults with IBS, ulcerative colitis, Crohn’s disease, or celiac disease (n = 20)	Open-label, multicenter, single-arm, pilot trial	4 g/d 2′-FL (n = 20)	-	6 weeks	- Improved IBS symptoms - Effect on gut microbiome composition - Effect on SCFA concentrations in feces
[69]	Healthy adults (n = 60)	Randomized, triple-blind, single-center, controlled trial	3 g/d 6′-SL (n = 30)	3 g/d maltodextrin placebo (n = 30)	12 weeks	- Safe and well tolerated - No effect on blood markers

2′-FL, 2′-fucosyllactose, 6′-SL, 6′-sialyllactose; HMO, human milk oligosaccharide; *H. pylori*, *Helicobacter pylori;* IBS, irritable bowel syndrome; LNnT, lacto-*N*-*neo*tetraose.

**Table 6:** Studied oligosaccharides and dosages in clinical trials.

Study Population	Study Product	Reference	Total HMO	Dosage								Other Bioactive Compounds
				2′-FL	3-FL	DFL	LNT	LNnT	3′-GL	3′-SL	6′-SL	
Healthy term infants	Infant formula (g/L)	[39]	0.2					0.2				
		[40]	0.2 1.0	0.2 1.0								GOS: 2.2 and 1.4 g/L
		[41]	0.2	0.2								FOS: 2.0 g/L
		[43]	1.5	1.0				0.5				
		[44]	0.25	0.25								*Bifidobacterium animalis* ssp. *lactis* (10^6^ CFU/g)
		[46]	1.5	1.0				0.5				*Limosilactobacillus reuteri* (CFU not specified)
		[47]	1.0	1.0					0.015			GOS: 7.2 g/L FOS: 0.8
		[49]	5.75	2.99	0.75		1.5			0.23	0.28	
		[50]	1.0	1.0								*Limosilactobacillus reuteri* (10^7^ CFU/g)
		[51]	1.5 2.5	0.87 1.45		0.10 0.14	0.29 0.48			0.11 0.18	0.14 0.24	
		[52]	5.75	3.0	0.8		1.5			0.20	0.30	
		[54]	1.0	1.0								FOS (not specified)
Infants with diagnosed or suspected allergies	Infant formula (g/L)	[55]	1.5	1.0				0.5				
		[56]	0.2	0.2								
		[57]	1.5	1.0				0.5				
		[58]	1.5	1.0				0.5				
Pre-term infants	Dietary supplement (g/kg/d)	[59]	0.374	0.34				0.034				
Healthy children	Young-child formula (g/L)	[60]	3.0	3.0								Lactoferrin, immunoglobulins, TGF-β, and milk fat
Obese and overweight children	Dietary supplement (g/d)	[61]	4.5	4.5 3.6				- 0.9				
Healthy adults	Dietary supplement (g/d)	[64]	5.0 10.0 20.0	5.0 10.0 20.0 - - - 3.33 6.67 13.32				- - - 5.0 10.0 20.0 1.67 3.32 6.67				
		[68]	4.0	4.0								
		[69]	3.0								3.0	
Adults with IBS	Dietary supplement (g/d)	[67]	5.0	4.0				1.0				
		[65]	5.0 10.0	4.0 8.0				1.0 2.0				
Adults with *Helicobacter pylori* infection	Supplement (g/d)	[62]	10.0							10.0		
		[63]	10.0 20.0							10.0 20.0		

Abbreviations: 2′-FL, 2′-fucosyllactose, 3-FL, 3-fucosyllactose; 3′-GL, 3′-galactosyllactose, 3′-SL, 3′-sialyllactose; 6′-SL, 6′-sialyllactose; DFL, difucosyllactose; FOS, fructooligosaccharides; GOS, galactooligosaccharides; HMO, human milk oligosaccharide; IBS, irritable bowel syndrome; LNT, lacto-*N*-tetraose; LNnT, lacto-*N*-*neo*tetraose; TGF-β, transforming growth factor β.

In the first group of studies, 2′-FL was added to infant formula alone [56] or in combination with other prebiotics such as GOS [40], fructooligosaccharides (FOS) [42,54], or a mixture of fermented GOS and FOS including 3′-GL [47]. It has also been combined with probiotic bacteria such as *Bifidobacterium animalis* ssp. *lactis* [44], and with *Limosilactobacillus reuteri* (*L. reuteri*) [50]. The 2′-FL concentration ranged between 0.2 and 1 g/L, and the study period varied from 35 days to 6 months. In one study, LNnT alone was added to infant formula at a concentration of 0.2 g/L for 16 weeks [39]. A mixture of 2′-FL and LNnT (1 and 0.5 g/L, respectively) was studied in healthy infants alone [43] or combined with *L. reuteri* [46]. Other studies assessed 2′-FL and LNnT in infants diagnosed with cows’ milk protein allergy (CMPA) for 1 week [55] or for 4 months plus a voluntary follow-up until 12 months of age [57,58]. In preterm infants, 2′-FL and LNnT mixtures were supplemented at a ratio of 10:1 (0.374 g/kg body weight/d) [59]. A mix of five HMOs (2′-FL, 3-FL, LNT, 3′-SL, and 6′-SL) with a total concentration of 5.75 g/L was tested in two infant studies for 4 months, with a voluntary follow-up at 6 months [49,52]. A similar blend containing DFL instead of 3-FL was tested at concentrations of 1.5 and 2.5 g/L in infant formula over 6 months [51].

In healthy children 1–2.5 years of age, 2′-FL was supplemented alone (3 g/L) or combined with immunoglobulins and lactoferrin in a formula drink over a period of 6 months [60]. Children with obesity and overweight between 6–12 years of age were provided with 2′-FL supplements alone (4.5 g/d) or combined with LNnT for a period of 8 weeks [61].

In healthy adults, one study assessed 6′-SL supplements of 3 g/d, administered as two doses 12 h apart, for 12 weeks [69]. Furthermore, 2′-FL and LNnT were administered to healthy adults individually and as 2:1 blends at doses of 5, 10, and 20 g/d for 2 weeks [64]. In a pilot study in adults with IBS, ulcerative colitis, Crohn’s disease, or celiac disease, 2′-FL was added to a nutritional formula at 3 g/d over 6 weeks [68]. A 4:1 blend of 2′-FL and LNnT was provided at doses of 5 g/d and 10 g/d in two studies involving adults with IBS [65,67]. Two studies assessed 3′-SL supplements in adults infected with *H. pylori*. One was an open-label study with a dose of 10 g/d administered as a bolus [62], whereas the other was a randomized, double-blind, placebo-controlled trial with doses of 10 and 20 g/d administered over 4 weeks [63].

### 3.4. Study Outcomes

#### 3.4.1. Safety

All the clinical trials discussed herein tested the safety of HMO supplementation by assessing adverse events linked with HMO intake and tolerance (Table 2, Table 3, Table 4 and Table 5). Depending on the study population and HMO products, the safety assessment was extended to consider additional outcomes.

Endpoints for the safety of HMO-supplemented infant formula included the measurement of growth outcomes according to WHO growth standards relative to a control formula and a breastfeeding reference group (Table 2 and Table 3). In preterm infants, safety was measured by non-inferiority to achieve full enteral feeding compared to a placebo group. The HMO supplementation resulted in a nonsignificant reduction of time until full enteral feeding by 2 days compared to the placebo group [57]. Studies in infants with allergies extended the safety profile to include hypo-allergenicity assessment and allergy symptom management. These studies confirmed the effective management of allergy symptoms following the intake of HMO-containing extensively hydrolyzed formula (EHF) [55,56,57] and amino acid-based formula (AAF) [58]. One study confirmed the hypo-allergenicity in a double-blind, placebo-controlled food challenge [55].

Studies in children and adults, where blood sampling was possible as a standard procedure, considered routine clinical parameters as part of the safety assessment [60,61,64,65,69]. Additionally, studies in adult IBS populations tested whether HMO supplements affected IBS symptoms [65,67,68]. All studies confirmed the safety of HMO supplementation and did not report adverse events in any age group or health status (Table 2, Table 3, Table 4 and Table 5).

#### 3.4.2. Growth

Growth was measured in all but one of the infant studies [59]. It was the primary outcome in 13 of 16 infant studies [39,40,43,46,47,49,50,51,52,54,56,57,58]. It was mostly assessed in terms of non-inferior weight gain per day in intervention groups compared to control formulas and breastfed groups throughout the intervention period. Other anthropometric parameters measured included length, head circumference, weight, body mass index, and interval gains, which were assessed either by comparison with reference groups or WHO child growth standards [39,40,42,43,44,46,47,49,50,51,52,54,57,58,59]. All studies reported age-appropriate growth following HMO supplementation and the absence of significant growth differences between intervention groups and controls (Table 2 and Table 3). One study in preterm infants with very low birth weights (<1700 g) reported a significantly higher length-for-age z-score in the HMO group compared to the placebo on days 14 and 21 before the full enteral feeding period. Mean head circumference gain in the HMO group was also significantly greater than that in the placebo group on day 21 before full enteral feeding [59].

#### 3.4.3. Tolerance

Tolerance outcomes were measured in all studies involving infants and children and in six of the adult studies. To evaluate tolerance in infants, most studies measured formula intake, gastrointestinal tolerance (flatulence, regurgitation, and vomiting associated with feeding), behavioral parameters (crying and fussiness), and stool characteristics, which are described in the next section (Table 2 and Table 3). HMO supplementation was consistently well tolerated by infants. No difference in intake between the formulas with and without HMOs [39,42,43,44,47,49,50,52,57,60]. Formulas with and without HMOs also invoked similar levels of flatulence [43,49,50,55], regurgitation [47,49], and vomiting associated with feeding [40,42,43,44,49,55]. There was also no effect on crying or fussiness [44,49,50]. One study reported similar frequencies of vomiting and flatulence in the HMO group compared to mixed-feeding and breastfeeding groups [46]. Single-arm trials that exclusively provided HMO-supplemented formula did not report feeding difficulties or gastrointestinal symptoms linked with HMO intake [56,58]. One study measured tolerance outcomes but has yet to report any results [51].

All studies that included infants with allergies measured tolerance outcomes. An EHF supplemented with 2′-FL and LNnT was well tolerated in infants and children diagnosed with CMPA [55]. The same formula was tested in another study, which showed that HMO supplementation had no effect on formula intake and digestive tolerance, with similar results in the HMO and control groups [57]. The addition of 0.2 g/L 2′-FL to an EHF was well tolerated when it was fed for 60 days. Parents reported improvements in tolerance outcomes (vomiting, regurgitation, constipation, and fussiness) and that allergy symptoms were resolved [56]. One study investigated an AAF supplemented with 2′-FL and LNnT in infants with moderate-to-severe CMPA who showed previous allergic responses to EHF. The HMO formula was well tolerated, with no gastrointestinal effects or feeding difficulties. Allergy symptoms improved significantly from enrollment, as assessed by physicians and parents. Two minor adverse events were associated with the study formula [58].

A formula supplemented with 3 g/L 2′-FL was well tolerated by children (1–2.5 years of age) with no effect on formula intake and no adverse events [60]. Similarly, supplements of 4.5 g/d 2′-FL alone or 2′-FL and LNnT in a 4:1 ratio were well tolerated by children (6–12 years of age) throughout the study period of 8 weeks, with no gastrointestinal discomfort. The number of adverse events was similar in the HMO and placebo groups. One participant reported mild gastrointestinal adverse events associated with the supplement [61].

In adults, tolerance was evaluated by filling questionnaires on gastrointestinal symptoms and discomfort, such as abdominal pain, indigestion, or reflux. HMO intake by healthy adults was well tolerated [64]. In adults with IBS, the severity and duration of bloating and abdominal pain declined significantly over 12 weeks when ingesting 5 g/d of a 4:1 mixture of 2′-FL and LNnT [67]. Similarly, another open-label trial in individuals with IBS and ulcerative colitis reported improved gastrointestinal quality of life after 6 weeks of receiving supplements of 4 g/d 2′-FL compared to baseline [68]. A 4-week study in which subjects received a 4:1 blend of 2′-FL and LNnT at doses of 5 and 10 g/d showed varying outcomes in adults with IBS. The HMO supplement was well tolerated and did not worsen prevalent gastrointestinal symptoms. The group receiving 10 g of the HMOs per day showed an improvement in gastrointestinal symptoms after 8 weeks but not after 4 weeks. The group that received 5 g HMO per day reported less flatulence after 4 weeks but not after 8 weeks [65]. In adults naturally infected with *H. pylori*, tolerability was evaluated based on adverse events. Supplements of 3′-SL did not increase the incidence of serious adverse events [62,63].

#### 3.4.4. Stool Characteristics

Stool characteristics were assessed in 18 trials, including 12 involving infants, two involving children, and four involving adults. Most studies assessed the frequency and consistency of stools using retrospective questionnaires. Infant formula supplemented with a mixture of 2′-FL, 3-FL, LNT, 3′-SL, and 6′-SL (5.75 g/L) influenced stool frequency and consistency compared to non-supplemented formula in two studies. The number of stools per day increased significantly, and the stool was significantly softer at specific time points, which represented a shift toward the stool characteristics of breastfed infants [49,52]. Additionally, the constipation dimension score was lower in the HMO group, and the supplemented formula resulted in predominantly yellow stools [52]. One study that supplemented infant formula with 1 g/L 2′-FL and 0.5 g/L LNnT for 6 months reported softer stools after 2 months. There were no effects on the average number of stools per day. Subgroup analysis of infants delivered by cesarean section revealed a lower incidence of infantile colic at 4 months [43]. The provision of HMO-supplemented AAF resulted in better-formed but less frequent stools over 4 months [58]. Other studies involving infants did not find effects on stool characteristics. In these cases, a formula was supplemented with 0.2–1 g/L 2′-FL [40,42,44,47,50,56], or an EHF was supplemented with 1 g/L 2′-FL and 0.5 g/L LNnT [55]. Similarly, HMO supplements had no effect on stool consistency in preterm infants but reduced stool frequency after 1 week of full-enteral feeding [59].

One study reported a lower number of days with hard stools in children receiving a formula supplemented with 2′-FL for 6 months compared to a control [60]. Supplements comprising 4.5 g/d 2′-FL and LNnT (4:1 ratio) over 8 weeks had no effect on stool consistency in children, as indicated by scores on the gastrointestinal symptom rating scale [61].

In healthy adults, regular stool characteristics were typically unaffected by 2-week HMO supplementation. Exceptionally, participants receiving 10 or 20 g/d of LNnT reported more frequent flatulence and harder stools, and those receiving 20 g/d of 2′-FL reported an increase in rumbling compared with baseline, as well as bloating, flatulence, and urgency to pass stools. These outcomes were considered minor and clinically irrelevant [64]. In adults with IBS, an open-label study reported a 20% decrease in hard stools and a 14% decrease in loose stools after 12 weeks of receiving 5 g/d of a 2′-FL/LNnT blend [67]. Another open-label study in individuals with IBS and other gastrointestinal conditions reported significant improvements in gastrointestinal quality of life and digestive symptoms following a 6-week intervention with 4 g/d of 2′-FL [68]. The provision of 5 or 10 g/d of a 4:1 mix of 2′-FL and LNnT in a double-blind study of IBS adults improved bowel habits in all intervention groups, with a significant reduction in abnormal stools compared to baseline [65].

#### 3.4.5. Infection Incidence

Ten trials involving infants and one involving children 1–2.5 years of age assessed the incidence of infections. The addition of 2′-FL alone to infant formula influenced the infection incidence in two studies. Post-hoc analysis showed that infant formula containing 0.2 g/L of 2′-FL reduced the number of respiratory infections to 4%, compared to 12% in the control, between the ages of 0 and 4 months [33]. In a similar trial of 2′-FL supplements, the frequency of infection fell from 23% to 8% [44]. In a trial of infant formula containing 2′-FL and LNnT administered for the first 6 months of life, parents retrospectively reported lower levels of infection and less frequent medication over the first year. The HMO-supplemented formula significantly reduced the risk of bronchitis in months 0–6 and 0–12, the use of antipyretics in months 0–4, and the use of antibiotics in months 0–12, compared to a non-supplemented formula [43]. The same HMO blend supplemented in EHF for infants with CMPA significantly reduced the frequency of upper respiratory tract and middle ear infections during the first year of life and significantly reduced the use of antipyretics between months 4 and 12 [57]. An evaluation of five HMOs (2′-FL, 3-FL, LNT, 3′-SL and 6′-SL) at a total concentration of 5.75 g/L reported lower visits to healthcare professionals between 0 and 4 months compared to infants that received the control formula (3% versus 12%) [52]. Other trials did not find evidence that HMOs reduced the number of infections in healthy infants [47,49,50,54] or preterm infants [59].

A trial in children receiving formula supplemented with 3 g/L 2′-FL assessed the incidence of upper respiratory tract infections as the primary outcome in the presence and absence of specific bioactive proteins. HMO supplementation did not have an effect on the incidence of upper respiratory tract infections or the duration of gastrointestinal infections. Prolonged upper respiratory tract infections, a higher number of days with fever, and episodes of cough and runny nose were reported for children that received formula with 2’-FL without bioactive proteins compared to the control group. The formula group with 2′-FL and bioactive proteins had a higher incidence of gastrointestinal infections compared to the control group [60].

#### 3.4.6. Gut and Nasopharyngeal Microbiome

Thirteen studies evaluated the effect of HMO supplementation on the gut microbiome: six in healthy-term infants, one in infants with CMPA, two in children, and four in adults. In one clinical study, infants received HMO-supplemented or standard formula until 6 months of age, followed by standard formula until 12 months [43]. The composition of the gut microbiome was analyzed in stool samples at 3 and 12 months in two follow-up studies. At 3 months, HMO supplementation shifted the microbiome composition closer to that of breastfed infants (compared to the control group), which featured a higher relative abundance of *Bifidobacterium* species and a lower relative abundance of *Escherichia* and *Streptococcus* species and the family *Peptostreptococcaceae*. The relative abundance of individual species and subspecies of bifidobacteria was not affected. Furthermore, fecal community types were clustered at the genus level. A fecal community type dominated by the family *Bifidobacteriaceae* was associated with the lower use of antibiotics between 0 and 12 months. At 12 months, no differences were found between the formula groups in terms of microbiome composition [45]. The association between microbiome composition and the occurrence of bronchitis and lower respiratory tract infections was studied in the second follow-up study. *Bifidobacterium longum* subsp. (*B.*) *infantis* was more abundant in infants that did not experience bronchitis or infection compared with those who did. A higher relative abundance of *Bifidobacterium* species at the expense of *Escherichia* species and the family Carnobacteriaceae was also reported at 3 months of age [48]. The same HMO blend added to an AAF was associated with gut microbiome changes at the phylum level (increase in Actinobacteria, decrease in Proteobacteria), genus level (increase in *Bifidobacterium*, *Akkermansia*, *Facealibacterium*, *Roseburia*, *Bacteroides,* and *Prevotella* species, decrease in *Enterococcus*, *Rothia*, *Klebsiella*, *Streptococcus,* and *Citrobacter* species), and species level (increase in *Akkermansia muciniphilia*, decrease in *Escherichia coli*). The relative abundance of butyrate-producing bacteria also increased, specifically infant-type *Bifidobacterium* (*B*.). *breve*, *B. bifidum*, *B. longum* ssp. *longum*, and *B. infantis* were significantly more abundant at 1 month of age. Overall, the relative abundance of HMO-utilizing bifidobacteria increased during the study period, while the relative abundance of Proteobacteria decreased [58].

Infant formula containing five HMOs (2′-FL, DFL, LNT, 3′-SL, and 6′-SL) at concentrations of 1.5 and 2.5 g/L also resulted in a gut microbiome profile closer to that of breastfed infant, particularly regarding the relative abundance of bifidobacteria [51]. The alpha diversity was reduced at 3 months for the 1.5 g/L supplement and at 6 months for the 2.5 g/L supplement. The relative abundance of total bifidobacteria was higher at 3 months for the 1.5 g/L supplement and at 6 months for both HMO groups. The relative abundance of *B. infantis* was higher at 3 months in both HMO groups and after 6 months in the 1.5 g/L HMO group. Notably, the relative abundance was already significantly higher at baseline for the 1.5 g/L HMO group compared to the control. The relative abundance of infant-type *Bifidobacterium* species was higher at 3 months for the 1.5 g/L HMO group and at 6 months for both HMO groups compared to the control. The relative abundance and prevalence of *Clostridioides* (*C*.) *difficile* were significantly reduced in both HMO groups compared to the control at 3 and 6 months. HMO supplementation also affected the relative abundance of the class Clostridia, the family Peptostreptococcaceae, and the genus *Lactobacillus* and *Streptococcus* relative to the control group, with the profile becoming more similar to that of the breastfed reference group [51].

In another study, supplements of 1 g/L 2′-FL again shifted the microbiome towards the profile of breastfed infants, particularly regarding bifidobacterial relative abundance [50]. At 3 months, the 2′-FL group was phylogenetically closer to the breastfed group than the control group. Furthermore, the relative abundance of the family *Peptostreptococcaceae* was reduced throughout the study period, whereas *Lactobacillus* species were less abundant at 2 months, and *C. difficile* was less abundant at 1 month [50]. Lastly, infant formula supplemented with 1 g/L 2′-FL and FOS versus a formula with 1 g/L GOS and FOS (concentration was not specified) resulted in a more efficient fucose utilization profile (closer to that of breastfed infants) and significantly higher glycosyl hydrolase activity associated with internal HMO utilization. The glycosyl hydrolase activity of the control group was lower, which was associated with extracellular HMO utilization. There were no differences in richness or Shannon diversity between the 2′-FL and control groups. At the phylum level, the relative abundance of Bacteroidetes was significantly higher in the non-supplemented group but was similar between the 2′-FL group and breastfed reference group [54].

In children between 1 and 2.5 years of age, formula supplemented with 3 g/L 2′-FL had no effect on the gut microbiome [60], but in children between 6 and 12 years of age, the addition of 4.5 g/d 2′-FL or 2′-FL and LNnT increased the abundance of *Bifidobacterium adolescentis* after 4 weeks [61].

In adults, two-week supplementation of 2′-FL, LNnT, or a 4:1 blend at total dosages of 5, 10, and 20 g/d generally modulated the intestinal microbiome, with the exception of the 20 g/d 2′-FL group. HMO supplementation increased the relative abundance of the phylum Actinobacteria, specifically due to the proliferation of Bifidobacteria [64]. HMO intake influenced the gut microbiome composition in adults with IBS in two trials. A 4:1 blend of 2′-FL and LNnT had no effect at 5 g/d, but 10 g/d significantly increased the abundance of Actinobacteria, *Prevotella* genus, and *Bacteroides* species after 4 weeks. After an additional four-week washout period, the microbiome changes did not persist [65]. In follow-up studies, the fecal and mucosal microbiomes showed an increase in the relative abundance of *Bifidobacterium* species (particularly *B. adolescentis* and *B. longum*) as well as the genus *Faecalibacterium* and the family Lachnospiraceae in fecal samples, and the genus *Blautia* in mucosal colonic biopsies samples [66]. Supplementation of 4 g/d 2′-FL at over 6 weeks increased the quantity of *B. longum*, *Faecalibacterium prausnitzii*, *Anaerotoruncus colihominis,* and *Pseudoflavonifractor* species in stool samples [68].

Two clinical trials tested the effects of HMO supplements on the nasopharyngeal microbiome. In the earlier study, infant formula was supplemented with 0.2 g/L LNnT, but the authors detected no impact on oropharyngeal colonization by *Streptococcus pneumoniae* [39]. In the more recent study, young children were provided with 3 g/L 2′-FL, which again had no effect on the nasal microbiome [60].

#### 3.4.7. Biomarkers in Fecal Samples

Ten studies analyzed fecal biomarkers. Four of these studies involved healthy-term infants, one involved infants with CMPA, one involved children, and four involved adults. Infant formula supplemented with 1 g/L 2′-FL reduced fecal pH and increased calprotectin levels at 2 months of age, while other fecal markers such as SCFA and secretory immunoglobulin A (sIgA) remained unaffected [50]. A formula supplemented with 2′-FL and LNnT changed the relative fecal abundance of acetate, butyrate, 5-aminovalerate, succinate, and fucosyl glycans compared to a non-supplemented formula at 3 months of age [48]. In addition, untargeted metabolomics revealed the modulation of amino acid *N*-acetylation and γ-glutamylation, as well as sphingolipid metabolism. The modulation of γ-glutamylation at 3 months correlated with a reduced risk of lower respiratory tract infections between 0 and 12 months [53]. AAF supplemented with 2′-FL and LNnT increased the fecal levels of acetate, butyrate, and propionate at 4–12 months [58]. A blend of 5 HMOs at doses of 1.5 and 2.5 g/L affected the fecal levels of sIgA, α-1-antitrypsin, and calprotectin. At 3 months, HMO supplementation resulted in increased sIgA and reduced α-1-antitrypsin levels compared to the control group. In the 2.5 g/L HMO group, the effect on sIgA persisted until 6 months. In the 1.5 g/L HMO group, calprotectin levels remained lower than in the control group for 6 months. The fecal pH was significantly lower in both HMO groups throughout the study period. Compared to the control group, the lactate concentration and the relative proportion of acetate were higher in the intervention groups, while the relative proportions of butyrate, isobutyrate, and isovalerate were lower [51].

In adults with IBS, supplementation of 4 g/d 2′-FL over 6 weeks increased total SCFA, acetate, and butyrate levels [68]. 2′-FL/LNnT supplementation in a 4:1 ratio at final concentrations of 5 g/L and 10 g/L affected fecal metabolites but not urine metabolites. Non-targeted metabolomic analysis identified two main differentiating metabolites (3-hydroxy-3-methylglutaric acid and N6-acetyl-l-lysine) that were modulated by HMO supplementation [66].

Two studies reported that HMO supplements had no effect on fecal biomarkers, one in which calprotectin levels in children were monitored for 8 weeks [61], and one in which SCFA (acetate, butyrate, and propionate), calprotectin, and sIgA levels were monitored in adults receiving 5, 10, or 20 g/d 2′-FL, LNnT, or both as a blend [64].

#### 3.4.8. Blood Analysis

Blood samples were collected in six studies, two involving healthy term infants, one involving children, and three involving adults. Blood samples were collected from healthy term infants 42 days after birth [40] and were analyzed for plasma cytokines and the ex vivo stimulation of peripheral blood mononuclear cells (PBMCs). Supplementation of 0.2 and 1 g/L 2′-FL significantly reduced plasma levels of pro-inflammatory cytokines (IL-1α, IL-1ra, IL-1β, IL-6, and TNFα) compared to the control group, resembling the profile of the breastfed group. 2′-FL supplementation also influenced the ex vivo stimulation of PBMCs, again resembling the breastfed group [41]. Another clinical trial collected blood samples at 6 months of age, but no results have yet been reported [51].

In children between 6 and 12 years of age, blood samples were analyzed for inflammation markers (TNFα, IL-1β, IL-6, IL-8, IL-10, and C-reactive protein), gut barrier integrity (LPS-binding protein, zonulin, and haptoglobin), and metabolic markers (adiponectin, leptin, resistin, soluble leptin receptor, ApoA1, ApoB100, free fatty acids, and ApoB48) before and after 8 weeks of exposure to HMOs. There were no significant effects [61].

Blood parameters were analyzed in healthy adults before and after 14 days of supplementation with 2′-FL, LNnT, or a blend at 5, 10, or 20 g/d, but there were no clinically relevant changes [64]. The provision of 3 g/d 6′-SL for 12 weeks also had no effect on clinical blood markers [69]. The non-targeted metabolomic analysis of plasma samples collected before and after 4 weeks of 2′-FL and LNnT intake at doses of 5 or 10 g/d in IBS patients revealed that the HMO supplements induced significant changes in the levels of asparagine and 7-methylguanine [66].

#### 3.4.9. Other Effects

One study in infants measured 2′-FL levels in plasma and urine at 6 and 17 weeks. Breastfed infants had significantly higher concentrations of 2′-FL than infants receiving 2′-FL supplemented formula at 1 and 0.2 g/L. However, the relative absorption rate was similar in the two groups (0.05–0.07% at 6 weeks and 0.02–0.03% at 17 weeks). The mean concentrations of 2′-FL in the urine were similar between the breastfed and 1 g/L 2′-FL formula groups but much lower in the 0.2 g/L 2′-FL formula group. In the control group, 2′-FL was not detected in plasma or urine samples [40]. The levels of 2′-FL and LNnT in urine and plasma were also studied in adult IBS patients after 4 weeks of receiving supplements of a 4:1 ratio of 2′-FL/LNnT at doses of 5 and 10 g/d. The authors detected 2′-FL at baseline and at 4 weeks in both groups and sample types but did not detect LNnT [66].

Two trials tested whether 3′-SL supplements can be used to treat *H. pylori* infections in adults. One was an open-label study in which subjects were provided with 10 g 3′-SL [62], and the other was a randomized, double-blind trial in which adults received 3′-SL supplements for 4 weeks at doses of 10 and 20 g/d [63], but neither of the supplements had any effect.

The expression of genes related to innate immunity and gut barrier function was measured in mucosal biopsies. The gene expression profiles were not influenced by the 2′-FL and LNnT blend supplements at doses of 5 and 10 g/d for 4 weeks compared to the placebo group. The same study evaluated the effects of HMOs on anxiety and depression, but no effects were observed [66].

## 4. Discussion

The aim of this systematic review was to summarize clinical trials that studied HMO supplementation in humans. We identified 31 publications that reported data across different populations, mostly infants but also children and adults. All trials reported that HMO supplementation was safe and well tolerated across all age groups and health states, and regardless of the HMO structures and doses applied. Indications for health benefits were also observed in the studies. In infants, many outcomes were shifted toward those of breastfed infants, such as the gut microbiome composition, stool characteristics, and blood and intestinal immune markers. The incidence of infections was also reduced in some trials. In children and adults, there was also evidence of microbiome modulation. Effects were observed for individual HMOs and blended at various doses.

In infants, HMO supplementation allowed age-appropriate growth and was not associated with any serious adverse events. In preterm and allergic infants, who represent especially vulnerable populations, HMO intake met hypo-allergenicity criteria according to recommended protocols [70,71]. Likewise, there were no safety or tolerance concerns in children and adults. The intake of 20 g/d HMOs as a bolus in adults resulted in mild adverse events [64]. Generally, the maximum tolerated amount of non-digestible carbohydrates is 5–20 g/d in adults, depending on the carbohydrate structure. Therefore, the reported adverse events are in line with expectations [72]. Moreover, the intake of such a high dose of HMOs as a bolus is unlikely to reflect any real-life situation.

Most of the clinical trials indicated changes in the gut-associated microbiome with HMO supplementation. The selective consumption of HMOs by species of *Bifidobacterium*, *Bacteroides,* and *Lactobacillus* provides them with a growth advantage [12,13]. In children and adults, HMO supplementation increased the abundance of bifidobacteria (Table 3 and Table 4), although the physiological relevance of this modulatory effect is unclear. A balanced gut microbiome is considered important for health, but the research community has yet to reach a consensus on how to define a healthy microbiome because the composition varies between healthy individuals [73]. In contrast, IBS is associated with gut dysbiosis, and HMOs may help to modulate the microbiome beneficially and manage IBS symptoms [34]. Supplementation of HMOs in subjects with IBS partially modulated the gut microbiome composition and influenced stool characteristics positively; however, findings require controlled replication in future studies [65,66,67].

The early-life microbiome development has a strong impact on both short-term and long-term health [74]. In infants, HMO supplementation changed the gut microbiome to resemble the profile of breastfed infants, including an increase in the relative abundance of bifidobacteria at the expense of pathogenic bacteria such as *C. difficile* [45,48,50,51,58]. HMO administration was associated with additional gut health outcomes that may be linked to the metabolic activity of the bacterial communities dominating the gut, including increased fecal content of SCFAs [48,51,58,68], reduced fecal pH [50,51], and modulation of microbial metabolic pathways [53]. HMO supplementation also promoted more frequent and softer stools [43,44,49,52,58]. Two studies reported changes in stool characteristics along with microbiome changes [43,58]. HMO-driven changes in the microbiome and in microbial metabolites such as SCFAs have been associated with a higher fecal water content, which may explain some of the observed changes [75]. The clinical significance of these outcomes has been discussed before because similar results were obtained for formulas supplemented with other prebiotics [76]. While the answer remains unclear, one of the main aims of supplementing infant formula with bioactive compounds is to shift the outcomes of formula-fed infants towards those of breastfed infants [77]. Hence, these results may be interpreted as positive.

Changes in the microbiome may also be associated with a reduced number of infections. Infants receiving HMO supplements suffered from less-frequent respiratory tract infections [33,43,58], were less likely to need attention from health professionals [52], and were less likely to experience otitis media [58]. Associations between fecal communities rich in bifidobacteria, microbial metabolic pathways, and reduced infections have been reported [45,53]. These outcomes may be mediated by a combination of mechanisms. In a *Bifidobacterium*-dominated gut environment, the growth of pathogenic bacteria is strongly discouraged. The microbiome may also support the immune system, either by direct interaction with immune cells [78] or by producing immunomodulatory metabolites [15]. In one clinical study, higher levels of sIgA, α-1-antitrypsin, and calprotectin were detected, along with an increase in the abundance of *B. infantis* [51]. In another study, 2′-FL was shown to reduce plasma levels of pro-inflammatory cytokines, but the microbiome composition was not investigated [41]. Lastly, HMOs can deflect pathogens by acting as decoy receptors and may modulate immune phenotypes, thereby supporting the immune system of the host [17]. Further studies are needed to elucidate to what degree the mechanisms suggested above are involved in the immune response triggered by HMOs.

HMOs are also proposed to have direct, microbiome-independent effects [79]. Two clinical trials detected supplemented HMOs in the plasma and urine of infants [40] and adults [66]. These results are in line with previous findings that a small percentage of ingested HMOs are absorbed, potentially in the small intestine via paracellular transport [79]. HMOs have also been detected in the circulation of breastfed infants [80,81,82]. Interestingly, only 2′-FL was detected in the plasma and urine in adults after 2′-FL and LNnT supplementation, perhaps because the latter is susceptible to degradation by host and microbial lactases [83]. The absorption of HMOs into the blood may facilitate their direct, microbiome-independent effects in the host. In the same infant cohort where 2′-FL was detected in blood and urine, the profile of inflammatory cytokines was affected similarly in the HMO group and the breastfed group [40,41]. However, these data are not yet conclusive evidence of direct, microbiome-independent effects. Future clinical research is needed to determine how certain HMOs are absorbed and what it means for systemic actions.

The clinical trials produced a wealth of data indicating that HMOs are modulators of the gut microbiome and are associated with additional health benefits. Potential health effects caused by supplemented HMOs have been discussed critically, especially in the context of infant formula. In some publications, it was stated that the number of existing clinical studies is too limited and does not allow conclusions about clinically relevant advantages of HMO supplementation [84,85]. However, these earlier reviews did not systematically assess the clinical evidence and omitted some of the studies discussed herein. Importantly, the primary outcomes of most clinical trials were safety and tolerability, with efficacy parameters as secondary or exploratory outcomes. This led to heterogeneous study designs, differing in the specific HMOs investigated, the dose, the food matrices that they were supplemented in, the age and health status of the enrollees, and the length of the study period. These differences make it more difficult to compare the results and identify structure–function relationships for individual HMOs and dose-dependent effects. Even so, exploratory indications for health benefits were evident from these studies, indicating the potential of HMO supplementation. Of note, not all infant studies measured changes in stool characteristics [40,42,44,47,50,55,56,59] nor infection incidence [47,49,50,54,59]. The diverse results may reflect the number of different HMOs tested and their relative and absolute doses. Studies reporting no effects typically involved only one or two HMOs at total doses of 0.2–1.5 g/L, whereas studies with more complex HMO mixtures and higher doses tended to achieve more significant effects.

Further clinical trials are needed to substantiate the beneficial effects of HMO supplementation, especially in the context of infant formula. They should be conducted over a longer study period and/or include follow-up investigations over longer time periods to assess potential long-term effects, such as immune development and neurodevelopment, using appropriate markers [86]. Future studies should also aim to determine which HMO structures and doses are needed to achieve relevant clinical outcomes. This may be particularly relevant for special populations, such as premature and allergic infants or adults with medical indications, who may benefit from HMO supplementation. One example of this concerns necrotizing enterocolitis (NEC), which is one of the most fatal disorders during neonatal care. The sialylated HMO disialyl-lacto-*N*-tetraose has been identified as a marker for reduced risk of NEC [87]. In addition, mixtures of 2′-FL and 6′-SL attenuated NEC inflammation in animal models [88]. HMO supplementation could be used to mitigate the risk of NEC, but the structure–function correlation and mode of action are still unclear.

**Figure 3:**
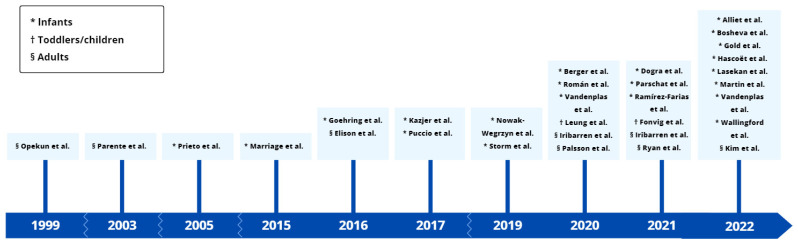
Time scale of published clinical trials that studied oral supplementation of manufactured human milk oligosaccharides in humans [39,40,41,42,43,44,45,46,47,48,49,50,51,52,53,54,55,56,57,58,59,60,61,62,63,64,65,66,67,68,69].

The number of human studies involving HMO supplementation has increased strongly over the past 20 years (Figure 3). At the time of writing, ten additional clinical trials were registered as ongoing in the ClinicalTrials.gov database, investigating HMOs as preventives or therapeutics (clinicaltrials.gov, accessed on 26 July 2023). In the time between the literature search and the preparation of this review, four clinical trial follow-up studies were published focusing on the effects of HMOs on the microbiome [89,90,91] or microbiome-derived markers [92]. This reflects that clinical HMO research is a rapidly evolving field with potential new insights to come out in the future.

This review was prepared in line with PRISMA guidelines [93]. The inclusion of studies that focused on the supplementation of manufactured HMOs as an intervention is one of the main strengths of this review. This approach allows for the identification of HMOs as the main causes of any observed effects. Moreover, there were no date restrictions in the literature search, which allows for a complete evaluation of available studies and a holistic overview of HMO supplementation. Limitations of this review include that the study protocol of the review was not prospectively registered and the lack of meta-analysis or synthesis methods to compare reported outcomes. Considering the limited number of studies and the heterogeneous study design, adequate meta-analysis is not yet possible, and the findings reported here should be interpreted with due care.

## 5. Conclusions

This systematic review provides an overview of the evidence for the health effects of HMO supplementation in humans based on clinical trials. The number of clinical trials involving HMO supplementation has increased greatly in the last few years and has resulted in diverse study designs. The clinical trials discussed here varied in terms of the HMO structures tested, the doses, the food matrices, the age and health of the enrollees, and the duration of the study. All clinical trials have confirmed the safety and tolerability of HMO supplementation in all study populations. Most studies also reported indications for health benefits associated with HMO intake. In infants, clinical outcomes such as the gut microbiome composition, stool characteristics, and immune responses were shifted towards those of breastfed infants. The ability of HMOs to modulate the gut microbiome was also evident in children and adults, although the clinical relevance is still unclear. Further evidence from well-designed clinical trials and preclinical experiments is required to substantiate the evidence for health benefits in different populations.

## Data Availability

No new data were created in this study. The data presented here are publicly available in the PubMed database and Cochrane Library.
